# HIV Controller CD4+ T Cells Respond to Minimal Amounts of Gag Antigen Due to High TCR Avidity

**DOI:** 10.1371/journal.ppat.1000780

**Published:** 2010-02-26

**Authors:** Benoît Vingert, Santiago Perez-Patrigeon, Patricia Jeannin, Olivier Lambotte, Faroudy Boufassa, Fabrice Lemaître, William W. Kwok, Ioannis Theodorou, Jean-François Delfraissy, Jacques Thèze, Lisa A. Chakrabarti, for the ANRS EP36 HIV Controllers Study Group

**Affiliations:** 1 Unité d'Immunogénétique Cellulaire, Institut Pasteur, Paris, France; 2 Institut National de la Santé et de la Recherche Médicale (INSERM) U802, Le Kremlin-Bicêtre, France; 3 Assistance Publique - Hôpitaux de Paris (AP-HP), Department of Internal Medicine and Infectious Diseases, Bicêtre Hospital, Le Kremlin-Bicêtre, France; 4 Université Paris-Sud, Le Kremlin-Bicêtre, France; 5 INSERM U822, Bicêtre Hospital, Le Kremlin-Bicêtre, France; 6 G5 Dynamiques des Réponses Immunes, Institut Pasteur, Paris, France; 7 INSERM U668, Equipe Avenir, Institut Pasteur, Paris, France; 8 Benaroya Research Institute at Virginia Mason, Seattle, Washington, United States of America; 9 INSERM U543, Pitié-Salpêtrière Hospital, Paris, France; Harvard Medical School, United States of America

## Abstract

HIV controllers are rare individuals who spontaneously control HIV replication in the absence of antiretroviral treatment. Emerging evidence indicates that HIV control is mediated through very active cellular immune responses, though how such responses can persist over time without immune exhaustion is not yet understood. To investigate the nature of memory CD4+ T cells responsible for long-term anti-HIV responses, we characterized the growth kinetics, Vβ repertoire, and avidity for antigen of patient-derived primary CD4+ T cell lines. Specific cell lines were obtained at a high rate for both HIV controllers (16/17) and efficiently treated patients (19/20) in response to the immunodominant Gag293 peptide. However, lines from controllers showed faster growth kinetics than those of treated patients. After normalizing for growth rates, IFN-γ responses directed against the immunodominant Gag293 peptide showed higher functional avidity in HIV controllers, indicating differentiation into highly efficient effector cells. In contrast, responses to Gag161, Gag263, or CMV peptides did not differ between groups. Gag293-specific CD4+ T cells were characterized by a diverse Vβ repertoire, suggesting that multiple clones contributed to the high avidity CD4+ T cell population in controllers. The high functional avidity of the Gag293-specific response could be explained by a high avidity interaction between the TCR and the peptide-MHC complex, as demonstrated by MHC class II tetramer binding. Thus, HIV controllers harbor a pool of memory CD4+ T cells with the intrinsic ability to recognize minimal amounts of Gag antigen, which may explain how they maintain an active antiviral response in the face of very low viremia.

## Author Summary

HIV infection, if left untreated, leads to the progressive disruption of the immune system, the destruction of the CD4+ T cell population, and the occurrence of multiple opportunistic infections. However, a small fraction of HIV-infected individuals (less than 1%) avoid these deleterious effects by spontaneously controlling HIV replication to very low levels in the absence of antiretroviral therapy. Emerging evidence indicates that these rare patients, named HIV controllers, contain HIV through a very active T cell-mediated immune response. In this study, we found that memory CD4+ T cells from HIV controllers had the capacity to respond to minimal amounts of antigen derived from the viral protein Gag. This property was intrinsic to controller CD4+ T cells, and resulted from the expression of T cell receptors (TCRs) with high avidity for a particular Gag peptide. The presence of high avidity CD4+ T cells may explain how HIV controllers maintain the antiviral immune response in constant alert, even though the amount of virus inducing this response is minimal. Based on this study, we propose that future candidate vaccines against HIV should induce high avidity memory CD4+ T cells, to mimic the rapid and persistent antiviral response characteristic of HIV controllers.

## Introduction

HIV controllers are rare individuals who spontaneously control HIV replication in the absence of antiretroviral treatment [1],[2]. HIV controllers harbor plasma viral loads that remain undetectable by conventional assays and cell-associated HIV DNA loads that are in the very low range, close to one log below those detected in patients receiving efficient antiretroviral therapy [3]–[5]. HIV controllers show a very low risk of progression to AIDS [3], emphasizing the importance of limited viral dissemination in maintaining a healthy status in the long term.

Emerging evidence indicates that controllers suppress HIV replication through a very active immunological process. HIV controllers harbor effector memory CD8+ T cells capable of rapidly killing infected autologous CD4+ T cells through a cytotoxic mechanism involving the upregulation of perforin and Granzyme B [6],[7]. Signs of immune activation are more prominent in HIV controllers than in efficiently treated patients, and include increased plasma LPS [8], increased expression of T cell activation markers [9], and increased propensity to secrete IFN-γ and MIP-1β upon polyclonal stimulation [10]. Longitudinal studies of efficiently treated patients who achieve undetectable viral load have shown a waning of cellular antiviral responses, which paralleled the progressive decrease in viral burden [11]. In contrast, HIV controllers maintain polyfunctional effector memory T cells with the capacity to secrete multiple cytokines [12]–[14]. How controllers maintain an active antiviral response in the long term in spite of a very low viral burden remains poorly understood.

One element contributing to the persistence of an active immune response may be the quality of the HIV-specific central memory (CM) compartment. CM T cells are thought to be responsible for the long-term maintenance of immune memory, due to their long half-life, high proliferative potential, and capacity to replenish the pool of effector and effector memory (EM) T cells that directly control pathogens [15]–[17] The progressive depletion of the CM CD4+ T cell compartment parallels disease progression in a simian model of AIDS [18]. CM CD4+ T cell functions, such as proliferation and IL-2 secretion, are impaired as early as the primary infection stage in progressive HIV infection [19]–[21], and are only partially recovered in efficiently treated patients [22],[23]. Chronic antigenic stimulation is thought to drive an accelerated differentiation of CM into effector CD4+ T cells, and thus contribute to T cell exhaustion. Importantly, CM CD4+ T cell numbers and functions are preserved in HIV controllers, who appear protected from this accelerated differentiation process [24],[25]. A recent study suggests that inactivation of pro-apoptotic molecules may contribute to the remarkable proliferative capacity of CM CD4+ T cells of HIV controllers, which can exceed that seen in healthy controls after non-specific stimulation [26].

We have previously shown that signs of CD4+ T cell immune activation could be detected in HIV controllers who nevertheless had an intact CM CD4+ T cell compartment, with preserved IL-2 secretion capacity and efficient proliferative responses [10]. How chronic immune activation was induced in controllers, and why it did not generally lead to accelerated CD4+ T cell differentiation and exhaustion remained unclear. To explore these issues, we tested the capacity of Gag-specific memory CD4+ T cell to differentiate *in vitro*, comparing primary CD4+ T cell lines derived from HIV controllers and efficiently treated patients with equivalent duration of infection. We found that HIV controller harbored a pool of memory CD4+ T cells able to differentiate into effector cells with high functional avidity for an immunodominant Gag epitope. This heightened sensitivity to Gag antigen could be explained by a high avidity interaction between the TCR and the peptide/MHC complex, as measured by class II tetramer binding. The capacity to mount a CD4 recall response in the presence of minimal amounts of Gag antigen may help explain how HIV controllers maintain a continuously activated antiviral response in spite of very low viremia.

## Results

### Rapid growth of CD4+ T cell lines from HIV controllers

Memory CD4+ T cell responses were compared in patients who spontaneously controlled HIV replication (HIC group, n = 17) and in patients who achieved viral control following successful antiretroviral therapy (HAART group, n = 20). Patients in both groups had viral loads <40 HIV RNA copies/ml plasma. The duration of infection and the CD4+ T cell count did not differ significantly between the two groups (Table 1).

**Table 1:** Virological and immunological characteristics of study subjects.

	HIV controllers (HIC, n = 17)	Treated patients (HAART, n = 20)	Viremic patients (VIR, n = 10)
Minimal duration of HIV infection, years	15 [10]–[21]	12 [7]–[20]	5 [2]–[9]
Duration of antiretroviral treatment, years	-	11 [7]–[13]	-
Viral load, HIV-1 RNA copies/ml plasma	<40	<40	44,268 [12,500–92,253]
CD4 count, cells/mm^3^ blood	741 [256–1207]	786 [422–1534]	430 [150–1087]
CD4+ T cell nadir, cells/mm^3^ blood	-	305 [11–589]	-

Median values [range] are reported.

We analyzed the properties of memory CD4+ T cell precursors by determining their capacity to generate CD4+ T cell lines specific for three immunodominant HIV-1 Gag peptides (Table 2). The peptides were chosen because of their broad immunodominance and their capacity to bind multiple HLA-DRB1 alleles [20], [27]–[30]. The frequency of response was determined by measuring the percentage of patients for whom viable CD4+ T cell lines (defined by a growth ratio >0.7 at day 14) could be obtained after stimulation with a Gag 20-mer peptide. PBMC from HIV-seronegative donors did not yield viable cell lines (not shown). 89 out of 90 cell lines obtained from HIV-seropositive donors proved peptide-specific, as indicated by a positive IFN-γ response measured in ELISPOT assay. The frequency of response to the most immunodominant peptide, Gag293, was remarkably high in both the HIC and HAART groups, with 94% and 95% of responders, respectively (Table 3). Responses to the second peptide, Gag263, were also frequent, with 82% responders in the HIC group and 77% responders in the HAART group. These findings confirmed that several CD4 epitopes in Gag could achieve strong immunodominance in patients with controlled HIV-1 infection. Interestingly, responses to the third peptide, Gag161, were more frequent in the HAART group than in the HIC group, with 91% versus 53% responders, respectively (P<0.05). We did not detect an association between the lack of response to Gag161 and particular HLA-DR genotypes. *Ex vivo* IFN-γ ELISPOT responses to the 3 Gag peptides were low, as expected for CD4 responses directed to single HIV peptides (Fig. S1). However, it was interesting to note that 7/13 Controllers had a detectable *ex vivo* response to Gag293 while only 1/13 Controller responded to Gag161 (P<0.05). This finding supported the notion of a higher frequency of Gag293-specific than Gag161-specific CD4+ T cells in Controller patients. Taken together, these observations suggested that the HIV controller status may be associated with a change in the immunodominance pattern of Gag CD4 epitopes.

**Table 2:** Peptides used to generate CD4+ T cell lines.

Peptide Name	Residues	Sequence	HLA DR genotype of tested patients
***HIV peptides***			
Gag293	293–312	FRDYVDRFYKTLRAEQASQE	Tested for all patients
Gag263	263–282	KRWIILGLNKIVRMYSPTSI	Tested for all patients
Gag161	161–180	EKAFSPEVIPMFSALSEGAT	Tested for all patients
***CMV peptides***			
pp65–509	509–523	KYQEFFWDANDIYRIF	HLA-DRB1*0101
″	″	″	HLA-DRB1*0301
pp65–281	281–295	IIKPGKISHIMLDVA	HLA-DRB1*0401
pp65–177	177–191	EPDVYYTSAFVFPTK	HLA-DRB1*0701
pp65–489	489–507	AGILARNLVPMVATVQGQN	HLA-DRB1*1101
pp65–41	41–55	LLQTGIHVRVSQPSL	HLA-DRB1*1501
pp65–489	489–507	AGILARNLVPMVATVQGQ	HLA-DRB5*0101

**Table 3:** Frequency of memory CD4+ T cell responses.

Peptides	HIV controllers (HIC)	Viremic patients (VIR)	Treated patients (HAART)
Gag293	94% (16/17)	70% (7/10)	95% (19/20)
Gag263	82% (14/17)	57% (4/7)	77% (10/13)
Gag161	41% (8/15)*	0% (0/6)***	92% (12/13)
pp65 CMV^a^	57% (9/14)	80% (4/5)	44% (8/18)

The frequency of obtention of a CD4+ T cell line with a positive IFN-g response is indicated in %.

The number of patients with a positive CD4+ T cell response reported to the number of patients tested is indicated in parentheses.

a total of responses to CMV peptides.

Differences in frequency were evaluated by Fisher's exact test.

*P<0.05 (HIC vs HAART).

***P<0.0005 (VIR vs HAART).

As controls, we generated CD4+ T cell lines specific for the CMV pp65 protein. Since the immunodominance pattern of CMV responses proved more variable than that observed for HIV-1, we optimized the generation of CMV-specific CD4+ T cell lines by choosing the pp65 peptide in function of the HLA DR genotype of the patients (Table 2) [31]–[33]. Using this strategy, close to half of the patients responded to pp65 peptides in both groups, which was consistent with CMV seroprevalence in the studied populations.

**Figure 1:**
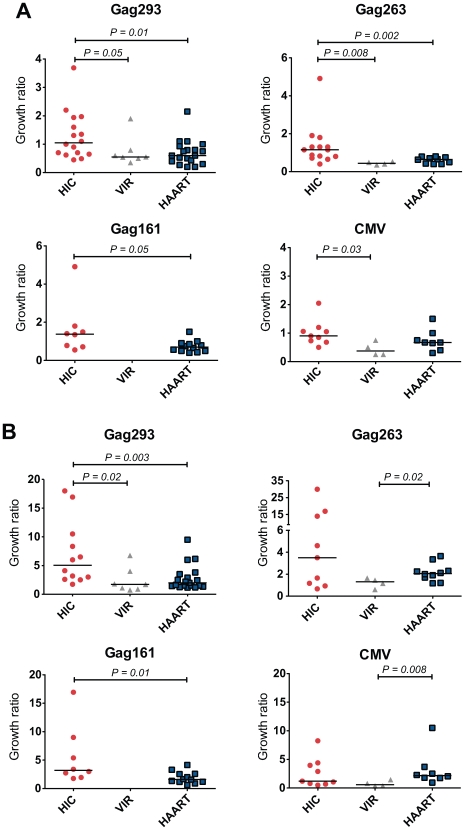
Rapid Growth of CD4+ T cell lines from HIV controllers. Growth of viable CD4+ T cell lines specific for Gag293, Gag263, Gag161 and CMV peptides was measured in groups of HIV controllers (HIC), viremic patients (VIR), and treated patients (HAART). The growth ratio, corresponding to the number of observed cells divided by the number of input cells, was evaluated at day 7 (A) and day 14 (B) of culture. Each symbol corresponds to one subject. Horizontal bars indicate medians. All statistically significant differences (P<0.05) evaluated by the Mann-Whitney U test are reported on graphs.

CD4+ T cell lines typically showed an initial loss of cells due to apoptosis, followed by growth due to multiplication of HIV- or CMV-specific cells. Measurement of the growth ratio at day 7 showed that viable CD4+ T cells lines from HIV controllers had a faster growth kinetics than those from treated patients, with a significant difference in response to the 3 Gag peptides but not in response to CMV peptides (Fig. 1A). Analysis of CD4+ T cell lines derived from a control group of untreated patients with HIV-1 viremia (VIR) showed limited growth capacity in all cases, consistent with the notion that active HIV-1 replication impaired the proliferative capacity of memory CD4+ T cells [19]. Measurement of growth ratios at day 14 (Fig. 1B) confirmed the rapid amplification of controller CD4+ T cell lines in response to Gag but not to CMV peptides. The clearest differences between the HIC and HAART groups were seen in response to Gag293 (P = 0.003), suggesting that HIV controllers harbored CD4+ T cell precursors with particularly good proliferative capacity in response to this immunodominant Gag peptide.

### Efficient differentiation of HIV controller CD4+ T cell precursors

**Figure 2:**
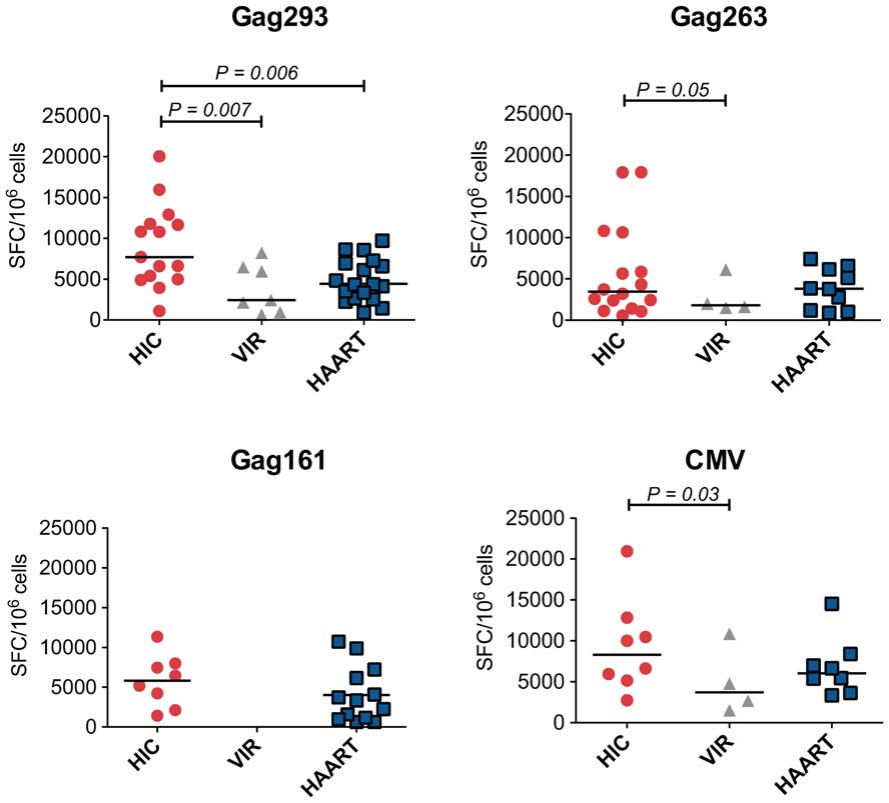
Increased IFN-γ production by CD4+ T cell lines from HIV controllers. Production of IFN-γ by CD4+ T cell lines specific for Gag293, Gag263, Gag161 and CMV peptides were compared between groups of HIV controllers (HIC), viremic patients (VIR), and treated patients (HAART). IFN-γ production was measured by ELISPOT assay on CD4+ T cell lines restimulated with peptide, and was expressed by the number of spot forming cells (SFC) per million cells. All measurements were made at CD4+ T cell line doubling time. Horizontal bars indicate medians. All statistically significant differences (P<0.05) evaluated by the Mann-Whitney U test are reported on graphs.

Analysis of IFN-γ production by ELISPOT at day 8 showed a prominent response in HIV controller CD4+ T cell lines (median SFC/10^6^ cells = 7,716), while most cell lines from treated patients remained negative. However, the fact that cell lines from treated patients had not yet entered the exponential growth phase could account for these differences. To compare CD4+ T cell lines at equivalent growth stages, all following measurements were made at doubling time (mean doubling time = 8 days in the HIC group, 13 days in the HAART group, and 14 days in the VIR group). In these conditions, IFN-γ production remained higher in the HIC group compared to the HAART group in response to the immunodominant Gag293 peptide (Fig. 2, P = 0.006). In control experiments, CD4+ T cell depletion abrogated the ELISPOT signal, confirming that IFN-γ was produced by CD4+ T cells, and not by the CD8+ T cells that may have escaped CD8 depletion (Fig. S2).

IFN-γ ELISPOT responses measured at doubling time were higher in the HIC group than in the VIR group for all peptide tested (Fig. 2). However, we and others have previously shown that when responses are measured *ex vivo*, which gives an evaluation of ongoing effector responses, CD4+ T cells from viremic patients produce as much IFN-γ as those of HIV controllers, while CD4+ T cells from treated patients have low IFN-γ production [10],[11],[22],[24]. The hierarchy of IFN-γ responses measured after proliferation of CD4+ T cell memory precursors is different, and rather reflects the capacity of these precursors to differentiate into cells with effector functions. We conclude that HIV controllers harbor memory CD4+ T cell precursors that can differentiate into efficient cytokine-secreting cells.

### Increased functional avidity of memory CD4+ T cells from HIV controllers

**Figure 3:**
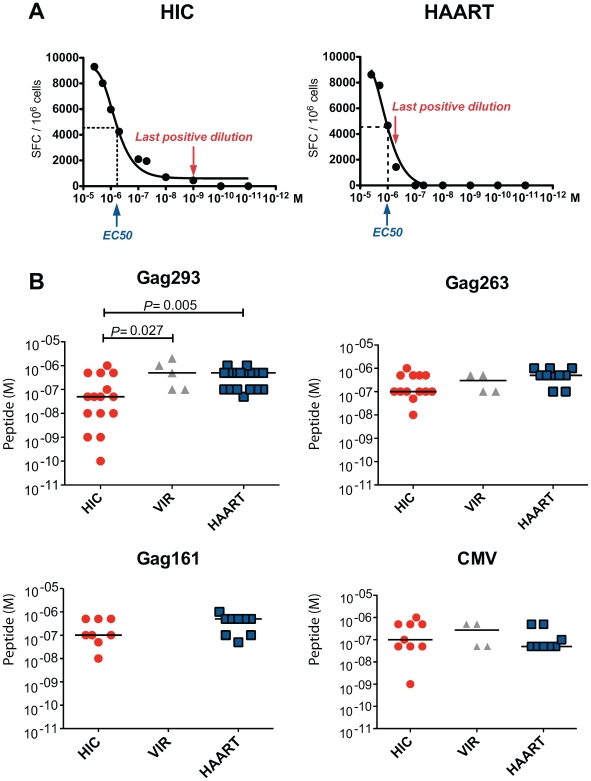
Increased functional avidity of memory CD4+ T cells from HIV controllers. The functional avidity was evaluated by IFN-γ ELISPOT assay in the presence of decreasing peptide concentrations (serial dilutions from 4×10^−6^ M to 10^−11^ M). For each peptide dilution, IFN-γ production was expressed as the number of spot forming cells (SFC) per million cells. The functional avidity was defined as the last peptide dilution that gave a positive IFN-γ response at least 2 fold above background level. (A) Representative examples of functional avidity measurement in response to Gag293 for one HIV controller (HIC) and one treated patient (HAART). The peptide dilution at half response (EC_50_) and the last positive peptide dilution are indicated. (B) Functional avidity of CD4+ T cell lines specific for Gag293, Gag263, Gag161 and CMV peptides were compared between groups of HIV controllers (HIC), viremic patients (VIR), and treated patients (HAART). All measurements were made at CD4+ T cell line doubling time. Horizontal bars indicate medians. All statistically significant differences (P<0.05) evaluated by the Mann-Whitney U test are reported on graphs.

To assess the sensitivity of memory CD4+ T cells to antigenic stimulation, we measured IFN-γ production in response to serial peptide dilutions (Fig. 3). We did not observe a significant difference in the dose of Gag293 peptide that induced a half-maximal ELISPOT response (median EC_50_ = 1.38 10^−6^ M in HIC vs. 1.55 10−6 M in HAART, P = 0.09). However, we observed that the shapes of the response curves differed, with a marked trailing end in the HIC group, suggesting the presence of a high avidity component within the responding CD4+ T cell population (see representative examples in Fig. 3A and Fig. S3). To extend this observation, we measured the last peptide concentration that gave a positive ELISPOT reading at least 2 fold above background. We verified that measurement of this concentration was reproducible in duplicate experiments (Table S1). Importantly, all measurements were carried out on CD4+ T cell lines at doubling time, to normalize for growth stage.

This analysis revealed that the functional avidity of CD4+ T cells recognizing the immunodominant Gag293 peptide was higher in HIV controllers (Fig. 3B). In the HIC group, 10 out of 15 patients tested had a positive ELISPOT reading at peptide concentrations ≤5 10^−8^ M, while in the HAART group only 1 out of 17 patients tested had a positive response at the same concentrations (P = 0.005). In contrast, functional avidities measured for Gag263, Gag161, and CMV peptides did not differ significantly between groups. These results pointed to a particular efficiency of CD4+ T cells specific for the immunodominant Gag293 in HIV controllers. Interestingly, the functional avidity of these cells correlated with the level of the IFN-γ response measured by ELISPOT assay at high peptide dose (R = −0.68, P<0.0001). Thus, CD4+ T cells responses to Gag293 appeared both sensitive and potent in the group of controller patients.

### Increased frequency of the HLA-DRB*0701 allele in HIV controllers

Patients were genotyped for the HLA DRB1 gene at 4 digit resolution. Table 4 reports the frequency of the most common HLA DRB1 alleles among the 34 HIV controllers and the 34 efficiently treated patients who were genotyped at initiation of the study. Interestingly, the frequency of the DRB1*0701 allele was 44% in the controller group and 18% in the treated patient group, which yielded a significant difference as measured by Fisher's exact test (P = 0.03). No other DRB1 allele showed significant differences. The frequency of the DRB1*0701 allele in the French population was reported to be 26% [34], close to that seen in the group of treated patients. In contrast, the frequency of DRB1*0701 appeared increased among HIV controllers.

**Table 4:** Phenotypic frequency of HLA DRB1 alleles in study subjects.

HLA DRB1 allele	HIV controllers (n = 34)	Treated patients (n = 34)
DRB1*0101	15% (5)	21% (7)
DRB1*0301	3% (1)	18% (6)
DRB1*0401	12% (4)	12% (4)
DRB1*0701 a	**44% (15)**	**18% (6)**
DRB1*1101	12% (4)	6% (2)
DRB1*1301	18% (6)	15% (4)
DRB1*1501	12% (4)	24% (8)

The frequency of individuals positive for a given HLA DRB1 allele is indicated in %.

The number of positive individuals is indicated in parentheses.

Differences in frequency were evaluated by Fisher's exact test.

Comparisons were made only for alleles present in 4 or more individuals in the controller group or the treated patient group.

a : a significant difference was detected for the DRB1*0701 allele (*P<0.05*).

To further explore the possibility that DRB7*0701 conferred an advantage in CD4+ T cell memory function, we compared functional parameters in DRB1*0701 positive versus DRB1*0701-negative individuals included in the study. We did not detect significant differences within the HIC group in terms of growth ratio, IFN-γ response, or functional avidity of CD4+ T cells following Gag293 stimulation. Within the HAART group, DRB1*0701-positive individuals showed lower IFN-γ responses (median SFC/10^6^ cells = 2458, n = 6) than DRB1*0701-negative individuals (median SFC/10^6^ cells = 6116, n = 9, P = 0.02). Taken together, these findings suggest that the DRB1*0701 allele may confer an increased chance to acquire a controller phenotype upon HIV infection, but that once the controller phenotype is established, the presence of the DRB1*0701 allele does not confer further benefit in terms of CD4+ T cell function.

### Rapid proliferation of Gag293-specific CD4+ T cells from HIV controllers

**Figure 4:**
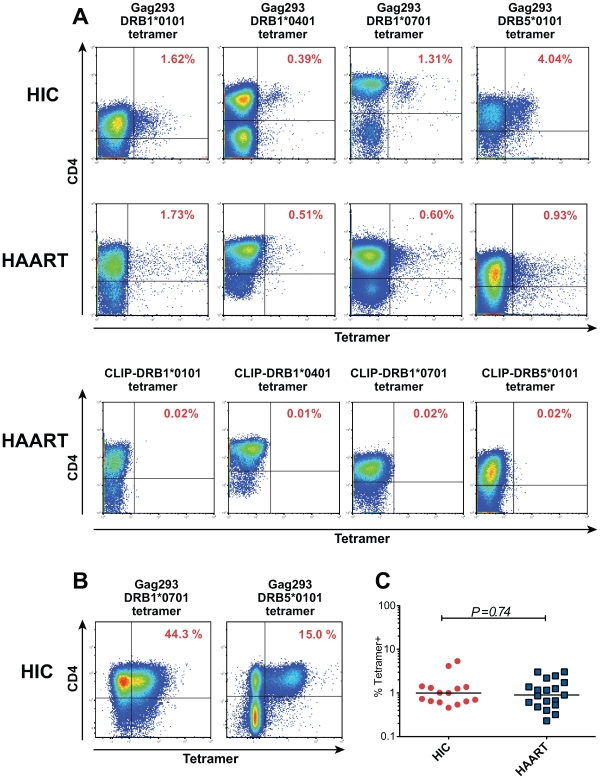
Characterization of HIV specific CD4+ T cells by MHC class II tetramer staining. (A) Representative examples of class II tetramer staining for Gag293-specific CD4+ T cells in cell lines at doubling time. Four distinct tetramers corresponding to alleles DRB1*0101, DRB1*0401, DRB1*0701 and DRB5*0101 were loaded with the Gag293 peptide and used to stain CD4+ T cell lines from HIV controllers (HIC) and treated patients (HAART) with matching HLA DR alleles. Negative controls corresponding to cell lines labeled with a DR-matched CLIP-loaded tetramer are shown for the 4 HAART cell lines. (B) Class II tetramer staining for Gag293-specific CD4+ T cells in cell lines from two HIV controllers (HIC) after 18 days of culture, showing the amplification of the tetramer+ population. (C) The percentage of Gag293-specific tetramer+ cells in CD4+ T cell lines at doubling time did not differ significantly between the HIC and HAART groups, as evaluated by the Mann-Whitney U test.

To further characterize the nature of Gag293-specific CD4+ T cells, we identified these cells through MHC class II tetramer labeling. Analysis of Gag293-specific CD4+ T cell lines revealed the presence of tetramer-positive (Tet+) cells for all the patients tested, confirming the antigen specificity of the cell lines (representative examples in Fig. 4A and 4B), and the capacity of the Gag293 peptide to bind multiple HLA-DR alleles [27]. At doubling time, the frequency of Tet+ cells was in the order of 1%. (Fig. 4A). The population of tetramer-negative (Tet−) cells may correspond to Gag293-specific cells restricted by an HLA-DR, -DP, or DQ allele distinct from that used in the tetramer, or to cells amplified through bystander effect. At later time points, the population of Tet+ cells could reach up to half of the CD4+ T cells (Fig. 4B), suggesting an efficient amplification of Gag293-specific cells restricted through HLA-DR. Comparison of the percentage of Tet+ cells at doubling time showed no significant difference between the HIC and HAART groups (Fig. 4C), which validated our normalization strategy. Namely, CD4+ T cell lines analyzed at equivalent growth stages contained equivalent numbers of peptide-specific cells, and could thus be usefully compared.

To determine the proliferative capacity of Tet+ cells, Gag293-specific CD4+ T cell lines were labeled with CFSE at doubling time and analyzed by flow cytometry 3 days later (Text S1, supplementary methods). The percentage of CD4+ Tet + cells that had divided (CFSE_lo_) was comparably high for the 3 HIC and 3 HAART cell lines analyzed (Fig. S4). The proliferative index, which represents the average number of divisions undergone by the population that divided, showed a trend toward higher values in the HIC cell lines. Interestingly, the difference became more apparent when the number of cells that had undergone 5 divisions or more was computed. In HAART cell lines, 6 to 7% of Tet+ cells had undergone 5 or more divisions, while in HIC cell lines these percentages were of 40%, 35%, and 18%. These data suggested that Tet+ cells from HIV controllers comprised a population endowed with an intrinsically high proliferative capacity and a short generation time.

### Diverse repertoire of HIV-specific CD4+ T cells in HIV controllers

**Figure 5:**
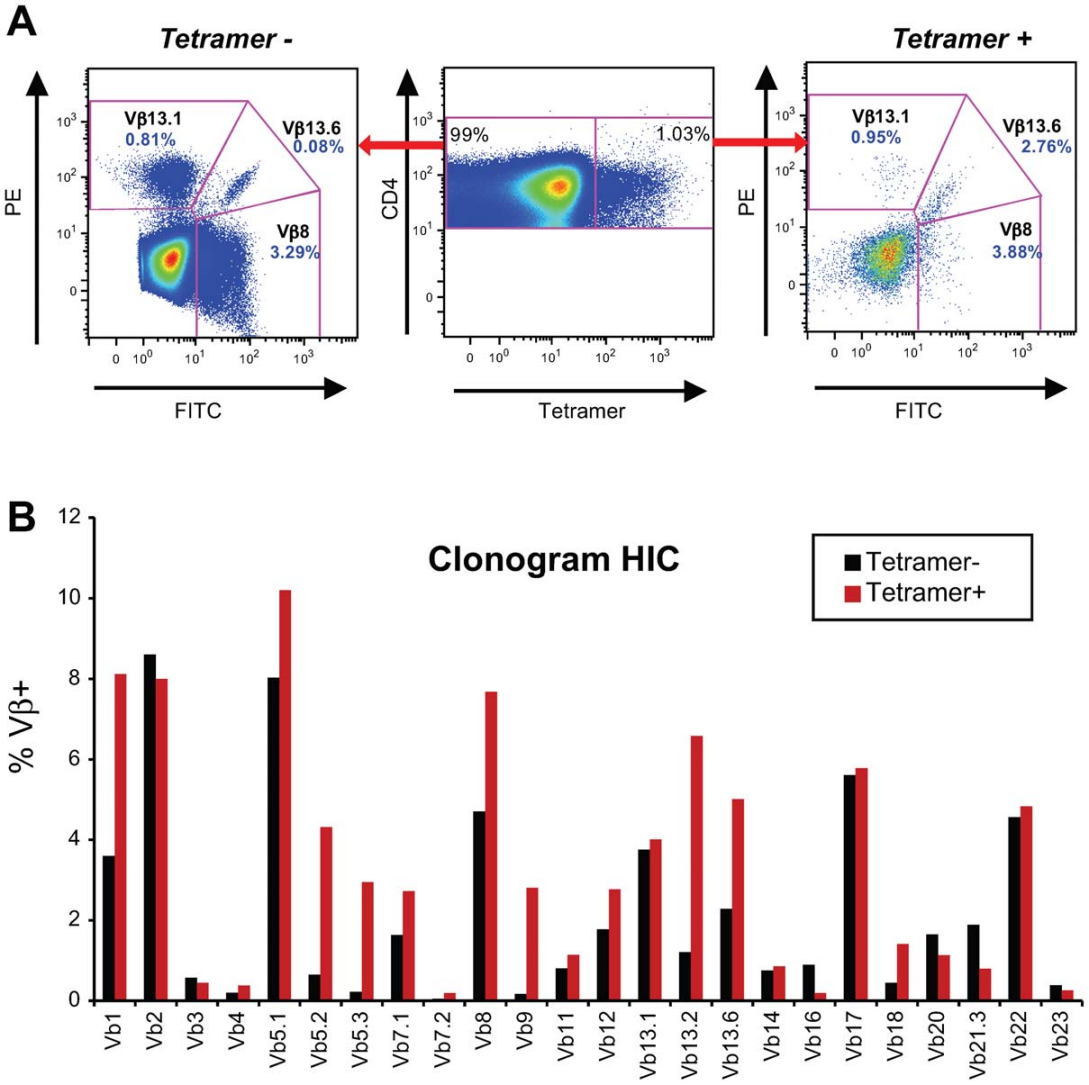
Gag293-specific CD4+ T cells have a diverse Vβ repertoire. (A) Example of TCR Vβ staining: comparison of three Vβ specificities in the tetramer-negative (left panel) and tetramer-positive (right panel) CD4+ T cell populations obtained for a Gag293-specific cell line from an HIV controller. In this case, Vb13.6 expression is amplified in the tetramer-positive population. (B) Example of Vβ repertoire analysis in an HIV controller CD4+ T cell line at doubling time. The clonogram indicates the percentage of CD4+ T cells labeled with a given Vβ-specific antibody within the tetramer-negative (black bars) and tetramer-positive (red bars) populations. The presence of multiple Vβ specificities within tetramer-positive population indicates that Gag293-specific CD4+ T cells have a diverse Vβ repertoire.

The TCR Vβ specificities of Tet+ and Tet− cells within CD4+ T cell lines were determined at doubling time by immunostaining with a panel of anti-Vβ antibodies (Fig. 5A and B). The TCR Vβ repertoire of Gag293-specific CD4+ T cells was diverse in both the HIC and HAART groups and varied depending on the individual. Table 5 lists the Vβ specificities showing an amplification within the Tet+ population, as defined by a ratio Tet+/Tet− ≥4 in a Tet+ population ≥2% of CD4+ T cells. The frequency of amplified Vβ populations is reported in supplementary Table S2. This analysis showed that Vβ1 was amplified in 3 out of 4 cell lines in the HAART group and in 2 out of 4 cell lines in the HIC group. Vβ9 and Vβ13.2 were also amplified in half of cell lines tested. These observations suggest that some Vβ chains may be preferentially selected in response to the Gag293 peptide. However, we did not detect a Vβ signature characteristic of the HIC group. Taken together, these data suggest that multiple clones contribute to the high avidity memory CD4+ T cell population in HIV controllers.

**Table 5:** TCR Vβ amplification ratios in Gag293-specific CD4+ T cell lines.

Vβ specificity	HIV controllers				Treated patients			
	# 1	# 2	# 3	# 4	# 1	# 2	# 3	# 4
1	-	5	5	-	7	-	6	6
2	-	-	-	-	-	-	-	-
3	-	-	-	-	-	-	-	-
4	-	-	-	26	-	-	-	-
5.1	-	-	5	8	-	-	-	18
5.2	7	15	-	-	-	-	-	-
5.3	13	7	-	-	-	26	-	-
7.1	-		7	-	-	21	-	15
7.2	-	-	-	-	-	-	-	-
8	-	-	-	-	-	-	-	-
9	16	7	-	-	-	6	-	14
11	-	-	-	-	-	-	-	-
12	-	-	39	-	-	-	7	-
13.1	-	-	-	-	25	-	-	-
13.2	5	4	-	-	-	-	5	25
13.6	-	9	4	-	20	-	-	-
14	-	-	-	-	-	-	-	-
16	-	-	40	-	-	-	-	-
17	-	-	-	-	-	-	-	-
18	-	-	-	-	-	-	-	-
20	-	-	5	-	-	-	-	-
21.3	-	-	-	-	-	-	-	-
22	-	-	5	-	-	-	-	-
23	-	4	-	8	-	20	-	-
**Total number of Vβ amplifications**	**4**	**7**	**8**	**3**	**3**	**4**	**3**	**5**

The ratio of tetramer-positive (Tet+) to tetramer-negative (Tet−) CD4+ T cells is given for each of the 24 Vβ specificities tested.

Only ratio ≥4 in populations of Tet+ cells ≥2% are reported.

### Increased TCR avidity of memory CD4+ T cells in HIV controllers

**Figure 6:**
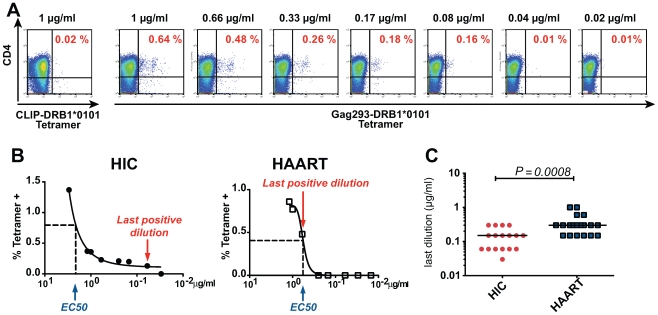
Increased TCR avidity of memory CD4+ T cells in HIV controllers. (A) The TCR avidity of Gag293-specific CD4+ T cells was determined by MHC class II tetramer staining in the presence of decreasing tetramer concentrations. An example shows the progressive decrease of Gag293-DRB1*0101 tetramer staining as a function of tetramer concentration in an HIV controller CD4+ T cell line at doubling time. (B) Representative examples of TCR avidity measurement in Gag293-specific CD4+ T cell lines from one HIV controller (HIC) and one treated patient (HAART). The tetramer dilution at half response (EC_50_) and the last positive tetramer dilution are indicated. (C) The TCR avidity was defined as the inverse of the last tetramer dilution that gave a positive staining at least 2 fold above control CLIP-tetramer staining. The TCR avidity of Gag293-specific CD4+ T cell lines was significantly higher in the controller group (HIC) than in the treated patient group (HAART) as evaluated by the Mann-Whitney U test. Horizontal bars indicate medians.

The high functional avidity of controller CD4+ T cells could result from increased avidity of the TCR for the pMHC complex, or from multiple factors that facilitate APC/T cell interactions or effector functions, including expression tuning of costimulatory molecules or efficiency of the IFN-γ secretion system [35]. To explore this issue, we set to directly test the avidity of the TCR/pMHC interaction. The TCR avidity was evaluated by measuring the percentage of Gag293-specific Tet+ CD4+ T cells detected as a function of decreasing class II tetramer concentrations, as described in reference [36] (Fig. 6A). The concentration measured at half-binding (EC_50_) did not show significant differences between the HIC and HAART groups. However, the shape of the binding curves differed between groups, with a persistence of detectable binding at low tetramer concentrations in the HIC group (Fig. 6B). The TCR avidity was measured by the inverse of the last concentration that gave a Tet+ staining at least 2 fold higher than control CLIP-tetramer staining. Comparison of Gag293-specific CD4+ T cells at doubling time showed that the TCR avidity was significantly higher in the HIC group than in the HAART group (Fig. 6C), indicating a difference in the nature of CD4+ T cell clones responding to the immunodominant Gag293 epitope. Thus, the high functional avidity of HIV controller CD4+ T cells could be explained, at least in part, by an intrinsic property of their TCR.

## Discussion

This study provides evidence that HIV controllers harbor a pool of high avidity memory CD4+ T cell precursors directed against an immunodominant Gag peptide. Memory CD4+ T cells specific for the Gag293 peptide were endowed with rapid growth potential and, importantly, with IFN-γ secretion capacity, suggesting that they would rapidly generate a pool of CD4+ T cells with effector function upon antigenic stimulation in controller patients. The high functional avidity of Gag293 specific cells points to their capacity of initiating recall responses in the presence of minimal amounts of HIV antigen. The high functional avidity could be explained, at least in part, by a high avidity interaction between the TCR and the cognate Gag293 peptide/MHC complex. Thus, the high sensitivity of controller CD4+ T cells to antigen appeared intrinsic, rather than dependent on the antigen presentation context or the cytokine milieu. The Vβ repertoire of tetramer-positive Gag293-specific cells proved diverse, suggesting that multiple clones contributed to the high avidity CD4 response in HIV controllers. This property may favor the long-term persistence of a high avidity response, since the presence of multiple clones reduces the probability of viral escape or of immune senescence. Taken together, these findings suggest that CD4 recall responses to Gag293 are rapid and efficient in the group of controller patients. We propose that the rapid triggering of recall responses may contribute to viral control. A rapid CD4+ T cell response upon occurrence of “viral blips” will keep the immune system in alert, provide immediate help for CD8+ T cells to exert efficient cytotoxic function, and possibly provide direct antiviral effector function [37]. This rapid recall response may help keep HIV-1 replication under a low threshold, and avoid the progressive undermining of the immune system associated with repeated viral replication episodes [38].

The presence of high avidity CD4+ T cells helps explain how HIV controllers maintain an active T cell response in the face of very low viremia. We and others have previously shown that the level of the specific CD4+ T cell responses in HIV controllers exceeds that seen in efficiently treated patients, even though both groups have very low antigenemia [10],[11],[22],[24]. The triggering of recall responses at very low antigenic load in the controller group may account for this difference. Emerging evidence suggests that the CD8+ T cell response may also be of high avidity in HIV controllers. In particular, individuals harboring the protective HLA-B27 allele frequently develop a high avidity response against the immunodominant KK10 CD8 epitope, the avidity of the response correlating inversely with viral load [39]. The fact that HIV controllers maintain antiviral CD8+ T cells with high cytotoxic potential in spite of their low viral load is also suggestive of a high avidity response [6],[7],[40]. Thus, both the CD4+ and the CD8+ T cell compartments may contribute to the high sensitivity to antigen characterizing antiviral responses in the controller group. Studies in mouse models of chronic viral infections have shown that efficient CD8 responses do not persist in the long term without CD4 help [41]. Therefore, a high avidity CD4 response may be essential in maintaining the quality of the CD8 response in low viremia conditions.

A correlate of a heightened sensitivity to HIV antigens may be a chronic level of immune activation, due to the recall of cellular responses upon each viral replication episode, however limited. Indeed, we have previously reported on signs of ongoing immune activation in the effector memory CD4+ T cell compartment of HIV controllers, as measured by the expression of HLA-DR, the downregulation of the IL-7 receptor, and the secretion of MIP-1β [10]. Other signs of activation include raised levels of LPS in plasma [8] and increased expression of HLA-DR within the HIV-specific CD8+ T cells [6] as compared to efficiently treated patients. These observations confirm the notion that viral control is achieved through an active immunological process. One should note that excessive chronic activation may be deleterious in the long term, as suggested by a trend toward CD4+ T cell decrease in controller patients with the highest degree of immune activation, even in the persistence of undetectable viral load [9]. It will be important in future studies to determine if such individuals show a decrease in T cell functional avidity, which may lead to more prolonged induction of recall responses to achieve viral control, and consequently to prolonged episodes of immune activation. On the other end of the activation spectrum, a few controllers appear to have low HIV-specific CD8 responses and a generally quiescent immune system [42]. This phenotype may result from a particularly successful viral control, with an antigenemia so low that it would not activate the high avidity memory T cell population for long periods of time. It also remains possible that non-T cell based, alternate mechanisms of viral control predominate in these rare individuals.

It was intriguing that HIV controllers responded less frequently to the Gag161 peptide than efficiently treated patients, while the quality of the CD4+ T cell response appeared generally better in the former group. This observation pointed to possible changes in the immunodominance pattern associated with the controller status. Responses to Gag161 may have become subdominant in the controller group due to competition by high avidity CD4+ T cells responding to other epitopes, including that present in the Gag293 peptide. Indeed, high avidity has been shown to sharpen immunodominance in mouse models [43]. The key mechanism appears to be the increased proliferative capacity of high avidity T cells, which progressively fill the memory T cell niche, a phenomenon accounting for the apparent avidity maturation of T cell responses over time [36],[44]. Importantly, in the present study, the duration of HIV-1 infection in the group of efficiently treated patients did not differ significantly from that in the controller group, with median of 12 (7–20) vs. 15 (10–21) years, respectively. Thus, a longer infection time was unlikely to account for the presence of high avidity CD4+ T cells in the controller group.

The CFSE analysis identified a population of Gag293-specific cells with high proliferative capacity in HIV Controller cell lines, which was consistent with the presence of a pool of high avidity CD4+ T cells. The number of divisions undergone by Tet+ cells was heterogeneous, with only a fraction reaching 5 generations and above. This may reflect a range of avidities for the Gag293 antigen, with only a fraction of Tet+ cells being endowed with high avidity and thus high proliferative capacity. This notion is also supported by the shape of the functional avidity curves, which suggests the presence of both high and low avidity populations within the pool of Gag293-specific cells from HIV Controllers. However, the high avidity component was absent in the Gag293-specific CD4+ T cell population from treated patients, independent of the method of analysis (functional avidity, tetramer avidity, or proliferation of Tet+ cells). One should note that the growth ratio of CD4+ T cell lines depended on the intrinsic proliferative capacity of specific cells but also on the frequency of these specific cells at the initiation of culture. We have previously shown that the frequency of p24 Gag-specific cells measured ex vivo by intracellular cytokine staining was approximately 3 fold higher in HIV controllers than in efficiently treated patients [10]. The analysis of ex vivo ELISPOT responses to the Gag293 peptide also showed a trend for higher values in the controller group. Thus, it is likely that both an increased precursor frequency and a higher proliferative capacity contributed to the efficient growth of CD4+ T cell lines from HIV controllers. Both properties may also contribute to the long term persistence of CD4 responses in these patients.

The genetic background may play a role in conferring a better ability to mount high avidity CD4+ T cell responses. The increased frequency of HLA DRB1*0701 in the controller group could suggest a beneficial effect of this allele on the development of anti-HIV CD4 responses. However, since we did not detect an association between the presence of HLA DRB1*0701 and the level or avidity of the CD4 response within the controller group, we speculate that this allele may play a role in initially facilitating viral control, rather than in maintaining high avidity CD4+ T cells. Alternatively, HLA DRB1*0701 may be in linkage disequilibrium with a protective MHC class I allele associated with viral control. A beneficial effect of the HLA DRB1*13 alleles on CD4 responses has also been suggested [13],[20], though we did not detect a significant effect in our study. It will be important to confirm these findings in cohorts of patients powered for large scale genetic studies. An intrinsic advantage in CD4+ T cell growth capacity may also promote efficient CD4 responses in controllers. Van Grevenynghe *et al.* [26] have reported an increased growth capacity of controller CD4+ T cell lines in response to polyclonal stimulation, as compared to cell lines derived from efficiently treated patients or even from healthy donors. These authors demonstrated a role for the activation of the PI-3 kinase pathway, and the resultant inactivation of the downstream apoptosis inductor FOXO3a, in this particular growth phenotype. We did note a trend for higher growth ratios in controller CD4+ T cell cultures in the presence of CMV peptides, even in the absence of a positive IFN-γ ELISPOT response (not shown). However, an increase in growth propensity only partially accounts for the CD4 response characteristics observed in HIV controllers. High avidity CD4+ T cells were directed against HIV but not CMV, pointing towards a selective advantage in the induction of anti-HIV responses.

The selection of high avidity gag-specific CD4+ T cells may result from a lower exposure to HIV antigens during the acute infection stage, when the repertoire of responding T cells is initially shaped. The few reported cases of acute HIV-1 infection followed by spontaneous viral control support the notion of a lower viral peak in patients who acquire a controller status [45]. Mouse models indicate that low antigen exposure is associated to the development of a high avidity response, since only the high avidity T cells receive sufficient signals through the TCR to proliferate in the long term [44],[46]. Such a scenario may predominate in patients who spontaneously control HIV replication. The presence of high avidity T cells may in turn stabilize the controller status by limiting viral replication episodes.

On the other hand, we cannot rule out that high avidity Gag-specific CD4+ T cells are selected but subsequently lost in progressor patients. Since high avidity CD4+ T cells are the first to respond in the presence of low HIV antigen amounts, they may be the first to get activated in the presence of replicating HIV, and may represent the initial wave of target cells available to the virus. HIV is known to preferentially infect HIV-specific cells [47], and among those it may well preferentially infect the most readily activated population. A recent report suggests that responses to several CD4 epitopes can be detected during the acute infection stage but are subsequently lost in progressor patients, which supports the idea of a rapid culling of the CD4 repertoire [21]. Another reason for the loss of high avidity CD4+ T cells may be senescence due to overstimulation by high antigenic loads in progressor patients. The observation that high avidity CD8 responses can be lost after acute HIV infection supports such a model [48]. An important area of future research will be to elucidate mechanisms that protect high avidity CD4+ T cells from depletion in HIV controllers.

In conclusion, this study provides evidence for the presence of high avidity CD4+ T cells directed against Gag in HIV controllers. It is remarkable that the distinctive properties of HIV-specific T cells in Controllers, including high proliferative potential [14],[24],[49], polyfunctionality [12],[13] and high cytotoxic capacity per cell [6],[7], are all known attributes of high avidity T cells [36],[40],[50],[51]. Thus, high avidity may underlie many of the characteristics of an efficient adaptive immune response against HIV. The presence of high avidity T cells has been associated with control of chronic viral infections in mice [50],[52], monkeys [53], and humans [54]. Since high avidity also confers long-term memory and rapid reactivation in presence of antigen [36],[44],[55], it represents a desirable property to be induced by candidate T cell vaccines against HIV.

## Materials and Methods

### Patients

HIV controllers (HIC group; n = 17) were recruited through the French “Observatoire National des HIV Controllers” established by ANRS. HIV controllers were defined as HIV-1 infected patients who had been seropositive for >10 years, had received no antiretroviral treatment, and for whom >90% of plasma viral load measurements were <400 copies of HIV RNA/ml. All HIV controllers included in the present study had current viral loads <40 copies/ml. Control groups included: (1) HAART group (n = 20): HIV-1 infected patients successfully treated with antiretroviral therapy for more than 5 years and with a viral load <40 copies of HIV RNA/ml; (2) VIR group (n = 10): viremic patients with viral loads >10,000 copies HIV RNA/ml. Viremic patients had been infected with HIV-1 for more than 1 year and had not received antiretroviral therapy. Patients from the HAART and VIR groups were recruited through the SEROCO-HEMOCO cohort and the Bicêtre hospital.

### Ethics statement

The study was promoted by ANRS under number EP36 and approved by the Comité de Protection des Personnes IDF VII under number 05–22. All participants gave written informed consent prior to blood sampling.

### Derivation of CD4+ T cell lines

PBMC from HIV infected patients were plated at 2×10^6^ cells per well in 24-well plates in the presence of one HIV-1 Gag or pp65 CMV peptide (10 µM) in RPMI 1640 supplemented with 10% human AB serum, 2 mM L-glutamine, 10 mM HEPES, 100 ug/ml penicillin/streptomycin, 0.5 µM AZT, 5 nM Saquinavir and 5 ng/ml recombinant IL-7 (Cytheris). The peptides used to stimulate the culture were highly purified 20-mers (>99% purity; PolyPeptide Laboratories). Recombinant IL-2 was added after 2 days to a final concentration 100 U/ml. Cell lines were restimulated with IL-2 every 2 days until the end of culture. Starting from day 7, cells were counted every day by trypan blue exclusion to determine the growth ratio (GR: observed number of cells/number of input cells at day 0). The CD8+ T cell population represented a median of 5.9% (range: 0–22.7%) of the CD3+ population. CD8+ T cells were depleted with magnetic beads (IMag particles, BD Biosciences) at doubling time (GR = 2), before performing functional assays. Less than 1% CD8+ T cells remained after CD8 depletion (Fig. S2A).

### ELISPOT assay

IFN-γ secretion by CD4+ T cell lines was evaluated by ELISPOT assay as previously described [56]. Briefly, 96-well nitrocellulose plates were coated with 1 µg/ml anti-human IFN-γ capture monoclonal antibody (Mabtech). Cell lines starved off IL-2 for 16 h were plated in duplicate at 30,000 cells/well in coated ELISPOT plates and incubated with 4 µM peptide for 24 h at 37°C. Wells were then washed, incubated with a biotinylated anti-IFN-g detection antibody (Mabtech), followed with alkaline phosphatase-labeled extravidin (Sigma-Aldrich), and with a chromogenic alkaline phosphatase-conjugated substrate. IFN-γ spot-forming cells (SFC) were counted with a Bioreader 4000 system (Bio-Sys). The ELISPOT response was expressed as SFC/10^6^ cells after subtracting background. Wells were counted as positive if the number of SFC was at least two times above background level. Functional avidity assays were carried out on all cell lines with ELISPOT responses >1000 SFC/10^6^ PBMC. ELISPOT responses were measured in response to serial peptide dilutions from 4×10^−6^ to 10^−11^ M, and the last dilution that gave a number of SFC at least two times above background was determined.

### MHC class II tetramer labeling

At initiation of the study, 34 HIV controllers and 34 treated patients were genotyped for HLA-DRB1. Patients were included in the study if their genotype matched at least one of the 6 HLA-DRB1 alleles available for MHC class II tetramer studies. This panel allowed the analysis of ≥70% of Caucasian patients [34]. PE-labeled tetramers for the DRB1*0101, DRB1*0301, DRB1*1501 and DRB5*0101 alleles were obtained through the NIH Tetramer Facility at Emory University. HLA-DRB1*0401, DRB1*0701, and DRB1*1101 biotinylated monomers were produced in insect cell cutures as previously described [57]–[59]. Monomers were loaded with 0.2 mg/ml peptide by incubation at 37°C for 72 h in the presence of 2.5 mg/ml n-octyl-b-D-glucopyranoside and 1 mM Pefabloc SC (Sigma-Aldrich). Peptide-loaded monomers were tetramerized using APC- or PE-conjugated streptavidin (eBioscience). To each tetramer loaded with the Gag293 peptide corresponded a control tetramer loaded with the CLIP peptide.

The class II tetramer labeling protocol was adapted from [38]. CD4+ T cell lines were incubated with 4 µg/ml class II tetramer for 90 min at 4°C in PBS-1% BSA buffer. Surface marker antibodies CD4-PerCP, CD3-AF750-APC, CD14-FITC (eBioscience), CD8-FITC, CD19-FITC (BD Biosciences), and the Aqua Live/Dead viability dye (Invitrogen) were added for the last 20 min of labeling. The percentage of tetramer-positive (Tet+) cells was measured in the live, CD3+, CD4+, CD8−, CD14−, CD19-gate. Events were acquired on a CyAn flow cytometer (Beckman Coulter, Fullerton, CA) and analyzed using the Flowjo software (Tree Star). Negative controls were obtained by staining with HLA-DR matched tetramers loaded with the CLIP peptide. To determine the avidity of the TCR/pMHC interaction, CD4+ T cell lines were incubated with decreasing concentrations of class II tetramer from 1 to 0.01 µg/ml. The avidity was defined as the inverse of the last concentration that gave a percentage of Tet+ cells at least 2 fold higher than CLIP-tetramer control values.

### TCR Vβ repertoire analysis

The TCR Vβ repertoire of HIV-specific CD4+ T cell lines was determined by co-staining cells with an MHC class II tetramer and a panel of Vβ-specific antibodies (IOt-Test Beta Mark TCR Vβ repertoire kit, Beckman Coulter), according to the manufacturer's instructions. The kit covered approximately 70% of human Vβ specificities. The Vβ nomenclature is that of Wei *et al.* [60]. A Vβ specificity was considered amplified when the Vβ frequency was increased at least 4 fold in the Tet+ compared to the Tet- population. A ratio of 4 was above the range of Vβ variation observed in the *ex vivo* repertoire of HIV-infected patients [61] and within the range of Vβ expansions induced by superantigens *in vitro* [62].

### Statistical analysis

Data are expressed as medians and range. Analyses were performed with the GraphPad Prism 5.0 software, using nonparametric statistical tests in all cases. Differences in variables between groups were analyzed with the Mann-Whitney U Test. Differences in percentages of response were analyzed with the Fisher's exact test. Correlations were analyzed with Spearman's coefficient R. All significant differences between groups (P<0.05) were reported on data plots.

## Supporting Information

Text S1 Supplementary methods and figure legends.
(0.03 MB DOC)

Figure S1 Analysis of *ex vivo* IFN-γ ELISPOT responses in PBMC from HIV controllers and efficiently treated patients. Production of IFN-γ by PBMC stimulated with Gag293, Gag263, and Gag161 20-mer peptides was measured in HIV controllers (HIC; n = 13) and treated patients (HAART; n = 6). IFN-γ production was measured by ELISPOT assay on PBMC plated at 10^5^ cells/well and stimulated with 4 µM peptide for 24 h. IFN-γ production was expressed as the number of spot forming cells (SFC) per million cells. Horizontal bars indicate medians. All statistically significant differences (P<0.05) evaluated by the Mann-Whitney U test are reported on graphs.
(0.31 MB EPS)

Figure S2 Control experiments demonstrating that IFN-γ production is mediated by CD4+ T cells. (A) Representative examples of the efficacy of CD8+ T cell depletion: The expression of CD4 and CD8 within the CD3+ T cell population was evaluated in Gag293-specific cell lines from two HIV controllers (HIC1 and HIC2). CD8+ T cells present in cell lines at doubling time (top row) were depleted with anti-CD8 coated magnetic beads. Less than 1% CD8+ T cells remained in the CD3+ population after depletion (bottom row). (B) Comparison of ELISPOT responses before and after CD8 of CD4 depletion: Gag293-specific CD4+ T cells lines from one HIV controller (HIC) and one treated patient (HAART) were evaluated at doubling time for IFN-γ production by ELISPOT assay. ELISPOT responses were measured on cell lines before depletion (No depletion), after depletion of CD8+ cells (CD8−), or after depletion of CD4+ cells (CD4−). The cellular concentration was normalized to 30,000 cells per well prior to ELISPOT analysis. IFN-γ production was expressed as the number of spot forming cells (SFC) per million cells. (C) Additional examples demonstrating that CD4 depletion abrogated the ELISPOT response: Gag293-specific cells lines from 3 treated patients were depleted either for CD8 or for CD4 prior to evaluation of IFN-γ production by ELISPOT assay.
(0.49 MB EPS)

Figure S3 Representative examples of functional avidity measurements in response to Gag and CMV peptides. (A) Functional avidity in Gag293-specific cell lines was evaluated at doubling time by ELISPOT assay. Representative examples are shown for 3 HIV controllers (HIC) and 3 treated patients (HAART). Functional avidity was measured by IFN-γ ELISPOT assay in the presence of decreasing peptide concentrations (serial dilutions from 4×10^−6^ M to 10^−11^ M). For each peptide dilution, IFN-γ production was expressed as the number of spot forming cells (SFC) per million cells. The functional avidity was defined as the last peptide dilution that gave a positive IFN-γ response at least 2 fold above background level. (B) Representative examples of functional avidity measurements in response to Gag263, Gag161 and CMV peptides. Cell lines from one HIV controller and one treated patient were analyzed in each case.
(0.64 MB EPS)

Figure S4 Analysis of the proliferative capacity of Gag293-specific cells. Gag293-specific CD4+ T cell lines from 3 treated patients (HAART) and 3 HIV controllers (HIC) were labeled at doubling time with CFSE. The Gag293-specific Tetramer+ CD4+ CD3+ population was analyzed 3 days later by flow cytometry. The number of generations was evaluated by the decrease in CFSE labeling. CFSE histograms were fitted with models that separated the populations into individual generations, using the FlowJo v8.8 software. The modeled generations are represented in dark blue, and the resulting curve fit is represented in red. “% divided” is the percentage of cells in the original sample which divided. “Proliferation index” is the average number of divisions undergone by the cells which divided (it does not take into account generation 0). Also reported in red is the percentage of cells that have undergone 5 or more divisions. This parameter highlights the presence of a population of Gag293-specific cells with high proliferative capacity in HIV controller cell lines.
(0.99 MB EPS)

Table S1 Reproducibility of functional avidity measurements.
(0.04 MB XLS)

Table S2 Frequencies of amplified TCR Vβ in CD4+ T cell lines specific for the Gag293 peptide.
(0.03 MB XLS)
