# Supplementary material for: HIV Controller CD4+ T Cells Respond to Minimal Amounts of Gag Antigen Due to High TCR Avidity
Source: PLoS Pathog. 2010 Feb 26;6(2):e1000780. doi: 10.1371/journal.ppat.1000780 (PMC2829066; doi:10.1371/journal.ppat.1000780)
Supplement: Text S1 — Supplementary methods and figure legends. (0.03 MB DOC) [file ppat.1000780.s001.doc]

Vingert et al.

**SUPPLEMENTARY TEXT**

**Figure S1: Analysis of *ex vivo* IFN-γ ELISPOT responses in PBMC from HIV controllers and efficiently treated patients**.

Production of IFN-γ by PBMC stimulated with Gag293, Gag263, and Gag161 20-mer peptides was measured in HIV controllers (HIC; n=13) and treated patients (HAART; n=6). IFN-γ production was measured by ELISPOT assay on PBMC plated at 10^5^ cells/well and stimulated with 4 µM peptide for 24h. IFN-γ production was expressed as the number of spot forming cells (SFC) per million cells. Horizontal bars indicate medians. All statistically significant differences (P<0.05) evaluated by the Mann-Whitney U test are reported on graphs.

**Figure S2: Control experiments demonstrating that IFN-γ production is mediated by CD4+ T cells**

(A) Representative examples of the efficacy of CD8+ T cell depletion: The expression of CD4 and CD8 within the CD3+ T cell population was evaluated in Gag293-specific cell lines from two HIV controllers (HIC1 and HIC2). CD8+ T cells present in cell lines at doubling time (top row) were depleted with anti-CD8 coated magnetic beads. Less than 1% CD8+ T cells remained in the CD3+ population after depletion (bottom row).

(B) Comparison of ELISPOT responses before and after CD8 of CD4 depletion: Gag293-specific CD4+ T cells lines from one HIV controller (HIC) and one treated patient (HAART) were evaluated at doubling time for IFN-γ production by ELISPOT assay. ELISPOT responses were measured on cell lines before depletion (No depletion), after depletion of CD8+ cells (CD8-), or after depletion of CD4+ cells (CD4-). The cellular concentration was normalized to 30,000 cells per well prior to ELISPOT analysis. IFN-γ production was expressed as the number of spot forming cells (SFC) per million cells.

(C) Additional examples demonstrating that CD4 depletion abrogated the ELISPOT response: Gag293-specific cells lines from 3 treated patients were depleted either for CD8 or for CD4 prior to evaluation of IFN-γ production by ELISPOT assay.

**Figure S3: Representative examples of functional avidity measurements in response to Gag and CMV peptides.**

(A) Functional avidity in Gag293-specific cell lines was evaluated at doubling time by ELISPOT assay. Representative examples are shown for 3 HIV controllers (HIC) and 3 treated patients (HAART). Functional avidity was measured by IFN-γ ELISPOT assay in the presence of decreasing peptide concentrations (serial dilutions from 4 x 10^-6^ M to 10-11 M). For each peptide dilution, IFN-γ production was expressed as the number of spot forming cells (SFC) per million cells. The functional avidity was defined as the last peptide dilution that gave a positive IFN-γ response at least 2 fold above background level.

(B) Representative examples of functional avidity measurements in response to Gag263, Gag161 and CMV peptides. Cell lines from one HIV controller and one treated patient were analyzed in each case.

**Figure S4: Analysis of the proliferative capacity of Gag293-specific cells**

Gag293-specific CD4+ T cell lines from 3 treated patients (HAART) and 3 HIV controllers (HIC) were labeled at doubling time with CFSE. The Gag293-specific Tetramer+ CD4+ CD3+ population was analyzed 3 days later by flow cytometry. The number of generations was evaluated by the decrease in CFSE labeling. CFSE histograms were fitted with models that separated the populations into individual generations, using the FlowJo v8.8 software. The modeled generations are represented in dark blue, and the resulting curve fit is represented in red. "% divided" is the percentage of cells in the original sample which divided. "Proliferation index" is the average number of divisions undergone by the cells which divided (it does not take into account generation 0). Also reported in red is the percentage of cells that have undergone 5 or more divisions. This parameter highlights the presence of a population of Gag293-specific cells with high proliferative capacity in HIV controller cell lines.

**Supplementary Methods**

**CFSE assay:**

CD4+ T cells lines were labeled with 0.35 µmol/l carboxyfluorescein succinimidyl ester (CFSE; Invitrogen) for 10 min at 37°C. After 2 washes in PBS supplemented with 5% FCS, the cell lines were cultured for 3 days in RPMI 1640 supplemented with 10% human AB serum, 2 mM L-glutamine, 10 mM HEPES, 100 μg/ml penicillin/streptomycin, and 100 U/ml IL-2. Cells were then labeled with Gag293-loaded class II tetramers as described in the Methods section and analyzed by flow cytometry. CFSE histograms were analyzed with the Proliferation platform of the FlowJo v8.8 software (Tree Star Inc.)
